# Prognostic value of early 18F-FDG PET scanning evaluation in immunocompetent primary CNS lymphoma patients

**DOI:** 10.18632/oncotarget.24706

**Published:** 2018-03-30

**Authors:** Rudy Birsen, Estelle Blanc, Lise Willems, Barbara Burroni, Marielle Legoff, Emmanuelle Le Ray, Sylvain Pilorge, Sawsen Salah, Aude Quentin, Benedicte Deau, Patricia Franchi, Marguerite Vignon, Laurence Mabille, Charles Nguyen, Yioula Kirova, Pascale Varlet, Myriam Edjlali, Edouard Dezamis, Khê Hoang-Xuan, Carole Soussain, Caroline Houillier, Diane Damotte, Johan Pallud, Didier Bouscary, Jerome Tamburini

**Affiliations:** ^1^ Paris Descartes University, Sorbonne Paris Cité, Paris, France; ^2^ Hematology Department, Cochin Hospital, Assistance Publique–Hôpitaux de Paris, Paris, France; ^3^ Department of Nuclear Medicine, Marie Lannelongue Hospital, Le Plessis Robinson, France; ^4^ Pathology Department, Cochin Hospital, Assistance Publique–Hôpitaux de Paris, Paris, France; ^5^ Ophtalmology Department, Cochin Hospital, Assistance Publique–Hôpitaux de Paris, Paris, France; ^6^ Jean Jaurès Hospital, Paris, France; ^7^ Radiotherapy Department, Curie Institute, Paris, France; ^8^ Department of Neuropathology, Sainte-Anne Hospital, Paris, France; ^9^ Department of NeuroImaging, Sainte-Anne Hospital, Paris, France; ^10^ Department of Neurosurgery, Sainte-Anne Hospital, Paris, France; ^11^ Department of Neurology 2-Mazarin, Groupe Hospitalier Pitié-Salpêtrière, Assistance Publique–Hôpitaux de Paris, Sorbonne Universités UPMC Universités Paris VI, IHU, ICM, Paris, France; ^12^ Hematology Department, René Huguenin-Institut Curie Hospital, Saint Cloud, France

**Keywords:** primary CNS lymphoma, PET scanner

## Abstract

Primary central nervous system lymphoma (PCNSL) is a rare topographic variant of diffuse large B-cell lymphoma (DLBCL). While prognostic scales are useful in clinical trials, no dynamic prognostic marker is available in this disease. We report here the prognostic value of early metabolic response by 18F-FDG PET scanner (PET) in 25 newly diagnosed immunocompetent PCNSL patients. Induction treatment consisted of four cycles of Rituximab, Methotrexate and Temozolamide (RMT). Based on patient's general condition, consolidation by high-dose Etoposide and Aracytine was given to responding patients. Brain MRI and PET were performed at diagnosis, after two and four cycles of RMT, and after treatment completion. Two-year progression-free (PFS) and overall survival (OS) were 62% and 74%, respectively for the whole cohort. Best responses after RMT induction were 18 (72%) complete response (CR)/CR undetermined (CRu), 4 (16%) partial response, 1 (4%) progressive disease and 2 (8%) stable disease. Response evaluation was concordant between MRI and PET at the end of induction therapy. Nineteen patients (76%) had a negative PET2. Predictive positive and negative values of PET2 on end-of-treatment (ETR) CR were 66.67% and 94.74%, respectively. We observed a significant association between PET2 negativity and ETR (*p* = 0.001) and longer PFS (*p* = 0.02), while having no impact on OS (*p* = 0.32). Two years PFS was 72% and 33% for PET2– and PET2+ patients, respectively (*p* < 0.02). PET2 evaluation may help to early define a subgroup of CR PCNSL patients with a favorable outcome.

## INTRODUCTION

Primary central nervous system lymphoma (PCNSL) is a rare central nervous system (CNS)-localized extra nodal variant of diffuse large B-cell lymphoma. Except for a marked decrease among HIV-positive patients since the introduction of highly active antiretroviral therapies, the rate of PCNSL has gradually increased in patients over 65 year-old these last decades without identified risk-factors [1]. PCSNL is reported as having a dismal prognosis, due to the seemingly limited blood-brain barrier crossing capacity of conventional lymphoma chemotherapies. However, recent advances highlighted that long-term remission and even cure could be achieved for a significant proportion of PCNSL patients using Methotrexate-based chemotherapy regimens and intensive consolidation strategies [2]. Current frontline management recommendations of PCNSL patients mostly rest on the results of prospective phase 2 trials and involve chemotherapy regimens using 3–8 g/m^2^ methotrexate for 4 to 8 infusions, with an overall response rate of 60–80% [3–6]. Formerly, radiotherapy was widely employed as a consolidation modality, but long-term follow-up of survivors revealed an excessive rate of neurotoxicity, leading the investigators towards radiation-free strategies [7]. Consolidation by high-dose chemotherapy and autologous stem cell transplantation (ASCT) shows promise and is actively investigated [5, 6, 8]. Other groups reported the feasibility and efficacy of high-dose chemotherapy without ASCT in this setting [9]. The main PCNSL prognostic factors identified so far are age and performance status, both integrated into the widely used International Extranodal Lymphoma Study Group experience (IESLG) and Memorial Sloan-Kettering (MSK) prognostic scores [10, 11]. The current guidelines for treatment follow-up are based on International PCNSL Collaborative Group (IPCG) radiographic response criteria [12]. In contrast to other lymphoma subtype, 18F-fluorodeoxyglucose (18F-FDG) positron emission tomography (PET) has barely been evaluated in PCNSL [13]. Here, we assessed the impact of early metabolic response evaluated by PET after two cycles of the Rituximab, Methotrexate and Temozolomide (RMT) regimen (PET2) in 25 consecutive newly-diagnosed immunocompetent PCNSL patients.

## RESULTS

Characteristics of the patients are provided in Table 1. Median age was 68. There were 9 men and 16 women, and most patients (56%) had a more than 1 performance status Eastern Cooperative Oncology Group (PS-ECOG) score. All patients had biopsy (either stereotaxic or following open surgery) and all tumors were diffuse large B-cell lymphoma. The initial PET excluded an extra-cerebral localization of lymphoma in all patients. One patient had a concomitant ocular involvement and one a concomitant CSF involvement at diagnosis.

**Table 1:** Characteristics of the patients at diagnosis

Characteristics	All (*n* = 25)	PET2– (*n* = 19)	PET2+ (*n* = 6)
Age, years, median (range)	68 (39–83)	68 (39–83)	69 (57–79)
Age < 60	8 (32%)	6 (32%)	2 (33%)
Male sex	9 (36%)	7 (37%)	2 (33%)
PS (ECOG)			
0	5 (20%)	4 (21%)	1 (17%)
1	6 (24%)	3 (16%)	3 (50%)
≥2	14 (56%)	12 (63%)	2 (33%)
IESLG risk group^*^			
Low	1 (4%)	1 (5%)	0 (0%)
Intermediate	12 (48%)	8 (42%)	4 (67%)
High	12 (48%)	10 (53%)	2 (33%)
MSK score			
1	2 (8%)	2 (11%)	0 (0%)
2	10 (40%)	6 (32%)	4 (67%)
3	13 (52%)	11 (58%)	2 (33%)
Positive CSF cytology^#,*^	1 (8%)	0 (0%)	1 (17%)
Ocular involvement^§,*^	1 (11,1%)	0 (0%)	1 (17%)
Germinal center type	7 (28%)	5 (26%)	2 (33%)
Non germinal center type	18 (72%)	14 (74%)	4 (67%)

PS: performance status; ECOG: Eastern Cooperative Oncology Group; IESLG: international extranodal lymphoma study group; MSK: Memorial Sloan-Kettering Cancer Center; CSF: cerebrospinal fluid; GC: germinal center; non-CG: non germinal center DLBCL subtypes. ^*^Data regarding CSF protein concentration were available in 14 patients. When missing, this data was considerate as elevated for the IESLG risk group assessment. ^#^available for 12 patients at diagnosis; ^§^available for 9 patients at diagnosis; ^*^Absence of CSF and ocular involvement were excluded in all patients without initial evaluation during follow up.

### Response to treatment (Table 2)

**Figure 1:**
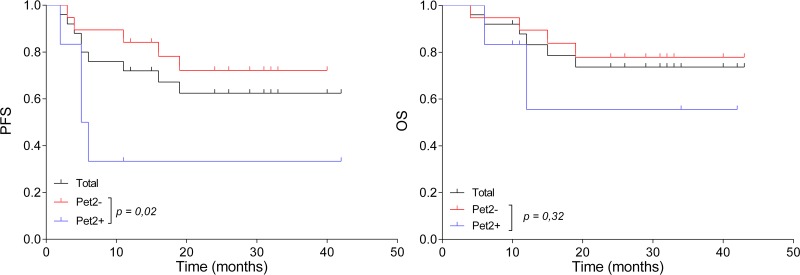
Survival based on PET2 evaluation Progression-free survival (PFS, **A**) and overall survival (OS, **B**) of the 25 patients who had a PET2 evaluation, based on PET positivity (PET+, *n* = 6) or negativity (PET–, *n* = 19).

Twenty patients (80%) completed the 4 cycles of RMT (19 responders and one progressive disease, PD). Among the 5 remaining patients, causes of treatment discontinuation were lymphoma-unrelated death (*n* = 1) and progression (*n* = 4). Seventeen patients in complete response (CR) following RMT underwent a consolidation therapy, by intensive chemotherapy (Etoposide and Aracytine (EA), *n* = 13), or by radiotherapy (23.4 Gy, *n* = 3 and 30.4 Gy, *n* = 1). With a median follow-up of 29 months (10–43 months), we observed 6 (24%) deaths, including 4 lymphoma-related and 2 lymphoma- or treatment-unrelated (suicide and pulmonary neoplasm). The two-year progression-free survival (PFS) and overall survival (OS) rates were 62% (CI 95%: 40–78%) and 74% (CI 95%: 50–87%), respectively (Figure 1). The best responses achieved during RMT induction were 18 (72%) CR/CR unknown (CRu), 4 (16%) partial response (PR), 1 (4%) PD, and 2 (8%) stable disease (SD). After treatment completion, 19 (76%) patients were in CR and 5 (20%) had PD. One (4%) patient was not evaluated (NE) due to lymphoma-unrelated death.

**Table 2:** Response to treatment

	During induction	Post-induction	After first line
**Complete response**	18 (72%)	16 (64%)	19 (76%)
**Partial response**	4 (16%)	0	0
**Stable Disease**	2 (8%)	0	0
**Progressive Disease**	1 (4%)	5 (20%)	5 (20%)
**MRI not done**	0	4 (16%)^*^	1 (4%)^‡^

Reported here are the best responses achieved during induction, responses after induction and at the end of the first line of treatment. Patients with stable or progressive disease during or after induction were switched to various salvage therapies. ^*^MRI not done due to treatment unrelated death (*n* = 1), protocol deviation (*n* = 3); ^‡^treatment unrelated death (*n* = 1).

### Comparison of PET and MRI results

A total of 57 concomitant PET and MRI evaluations were performed. We found a strict correlation between PET and MRI for CR (*n* = 38) and SD/PD (*n* = 4) assessment. In patients with MRI-based PR evaluation (*n* = 7), PET was found positive and negative in 2 and 5 cases, respectively. In MRI-defined CRu (*n* = 9), PET was negative in 8 cases and positive in one.

### PET2 analysis

**Figure 2:**
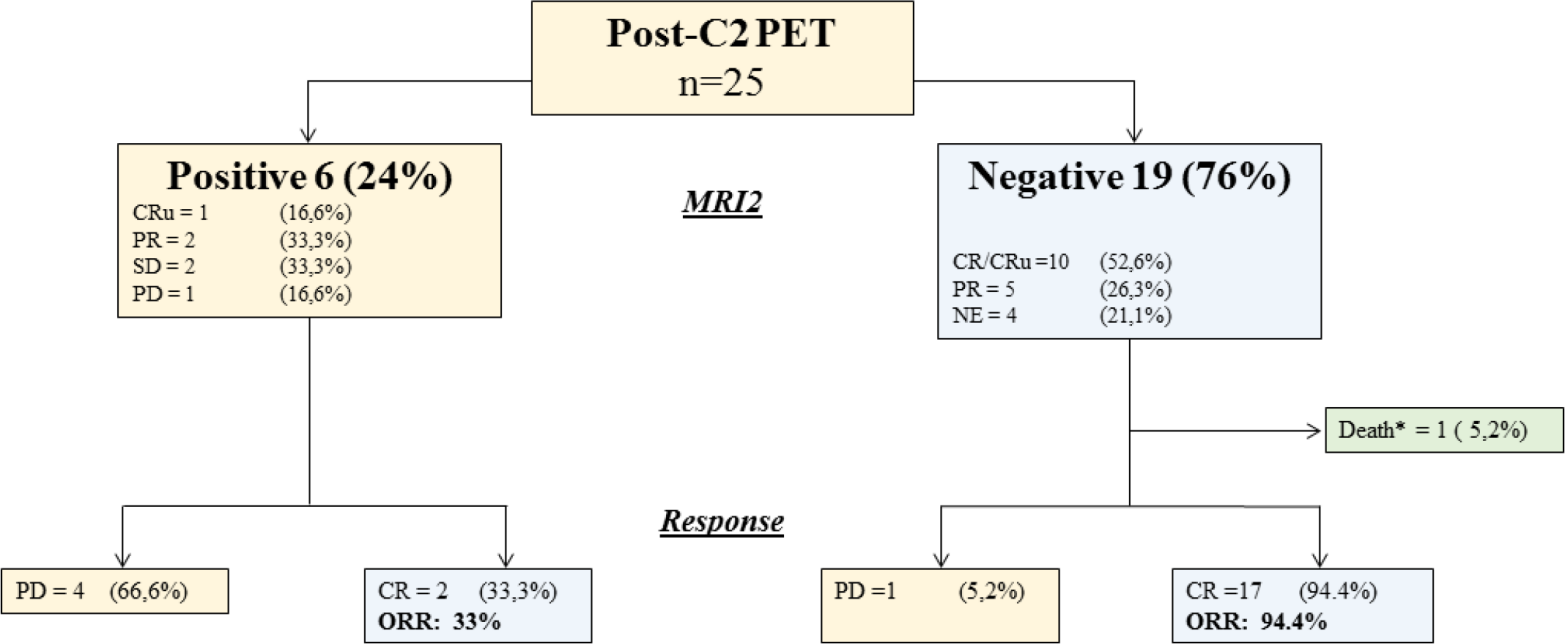
Response and outcome based on PET2 evaluation Twenty-five patients had a PET2 evaluation. Nineteen were negative and 6 positive. Correlation to post-C2 (MRI2) and end-of-treatment (MRIe) MRI evaluation is provided. CRu: complete response unconfirmed; PR: partial response; SD: stable disease; PD: progressive disease; NE: not evaluable; CR: complete response; ORR: overall response rate; death^*^: death from lymphoma-unrelated cause.

Six patients (24%) had a positive PET2 (PET2+), and concomitant MRI showed CRu (*n* = 1), PR (*n* = 2), SD (*n* = 2) and PD (*n* = 1). Nineteen patients (76%) had a negative PET2 (PET2–), among whom we observed 10 CR/CRu, 5 PR and 4 NE by MRI (Figure 2 and Table 3). Among PET2+ patients, four (66%) had a progressive disease while the two remaining achieved a CR. Among PET2- cases, a single patient had a localized intraocular evolution neither detected by PET nor MRI; and one patient in CR died from lymphoma-unrelated cause. Predictive positive and negative values (PPV/PNV) of PET2 on end-of-treatment CR were 66.67% (CI 95%: 33.34–88.89%) and 94.74% (CI 95%: 75.61–99.05%), respectively, without significant impact of MRI imperfections as a reference test for CR. Moreover, accuracy of PET2 was 88% (CI 95%: 68.78–97.45%), suggesting that PET2 adequately predicted outcome in most cases in our study. During the follow-up, two PET2–patients relapsed, and another died from lung cancer while remaining in CR. We evaluated several parameters for correlation with MRI end-of-treatment response (ETR) and survival. Age over 60, sex, ECOG, Memorial Sloan Kettering (MSK), international extra nodal lymphoma study group (IESLG) prognostic scores, and tumor characteristics including topography (deep, multiple lesions) and histological subtype (germinal center (GC) or non GC) had no incidence progression free and overall survival (Table 4). Interestingly, we observed a significant association between PET2 and ETR (*p* = 0.001) and PFS (*p* = 0.02), while PET-defined response had no impact on OS (*p* = 0.32). Two years PFS was 72% (CI 95% 45–87%) and 33% (CI 95% 5–67%) for PET2-and PET2+ patients, respectively (Figure 1).

**Table 3:** Response assessment by PET and MRI

Patient	Age	PS	Cs	PETd	MRI2	PET2	MRI4	PET4	MRIe	PETe	FU	Events
**1**	64	0	EA	19,6	CRu	N	CR	N	CR	N	43+	RE, SAL, ASCT
**2**	39	1	EA	14	PR	N	ND	N	CR	N	32+	-
**3**	77	2	EA	29,2	PR	N	CR	N	CR	N	29+	-
**4**	60	0	EA	r	CR	N	CR	N	CR	N	29+	-
**5**	69	2	EA	22,6	CRu	N	CR	N	CR	N	40+	-
**6**	78	3	-	50	ND	N	Cru	N	CR	N	33+	-
**7**	66	2	EA	21,6	PR	N	ND	N	CR	N	31+	-
**8**	78	3	RT	8,5	PR	N	ND	ND	CR	ND	26+	-
**9**	60	1	EA	19	ND	N	Cru	N	CR	N	29+	-
**10**	80	0	-	r	CR	N	CR	N	ND	ND	15	Ocular progression, SAL, LRD
**11**	77	0	RT	23,2	CRu	N	CR	N	CR	N	24+	-
**12**	75	2	RT	8,3	ND	N	CR	N	CR	N	19	Death (lung cancer)
**13**	79	2	RT	24,6	ND	N	CR	N	CR	N	24+	-
**14**	59	2	EA	32,9	CRu	N	CR	N	CR	N	19+	-
**15**	83	3	-	r	CR	N	CR	N	CR	N	32+	Pulmonary embolism
**16**	47	2	EA	24,7	PR	N	CR	N	CR	N	15+	-
**17**	58	2	EA	r	CR	N	CR	N	CR	N	11	RE, LRD
**18**	63	1	EA	r	CR	N	CR	N	CR	N	11+	-
**19**	68	3	-	21,6	CRu	N	ND	N	ND	ND	4	Death (suicide)
**20**	71	1	EA	13,8	PR	P	Cru	N	CR	N	42+	-
**21**	57	3	-	34,7	PR	P	PD	P	ND	ND	34+	SAL, ASCT
**22**	78	2	-	32,4	SD	P	ND	ND	ND	ND	6	LRD
**23**	79	1	-	18,8	SD	P	ND	ND	ND	ND	12	LRD
**24**	66	1	-	33,2	PD	P	ND	ND	ND	ND	10+	SAL, ASCT
**25**	57	0	EA	63	CRu	P	CR	N	CR	N	11+	-

PS: ECOG performance status; Cs: consolidation; PETd: PET at diagnosis (SUVmax); MRI2: MRI after 2 RMT cycles; PET2: PET after 2 RMT cycles; MRI4: MRI after 4 cycles; PET4: PET after 4 cycles; MRIe: MRI at the end of treatments; PETe: PET at the end of treatments; FU: follow-up (months); r: complete surgical resection; ND: not done; EA: etoposide cytarabine; ASCT : autologous stem cell transplantation; RT: radiotherapy; SAL : Salvage therapy; SD: stable disease; PD: progressive disease; PR : partial response; CR: complete remission; CRu: complete remission unknown; N: negative; P: positive; +: alive; RE: relapse; LRD: lymphoma-related death.

**Table 4:** Correlation between patient variables and progression or death

Variable	*N*	PD	Chi^2^	*p*-val	D	Chi^2^	*p*-val
Age < 60	8	2	0,5	0,47	1	0,72	0,39
Age > 60	17	7			5		
Male	9	3	0,21	0,64	1	0,87	0,35
Female	16	6			2		
ECOG PS 0–1	11	4	0,06	0,79	2	0,39	0,52
ECOG PS 2–4	14	5			4		
Normal LDH	19	6	0,78	0,38	4	0,34	0,55
High LDH	6	3			2		
MSK 1	2	0	0,95	0,62	0	0,74	0,69
MSK 2	10	4			2		
MSK 3	13	5			4		
IESLG Low	1	0	0,88	0,64	0	0,39	0,82
IESLG Int.	12	5			3		
IESLG High	12	4			3		
Single lesion	12	4	0	0,98	2	0,37	0,53
Multiple lesions	13	5			4		
Superficial lesion	5	1	0,23	0,62	1	0,06	0,79
Deep lesion	20	8			5		
Resected	5	2	0,1	0,74	2	0,94	0,32
Non resected	20	7			4		
GC	7	3	0,05	0,85	2	0,05	0,81
Non GC	18	6			4		
PET2–	19	5	5,1	0,02	4	1.15	0.32
PET2+	6	4			2		

The following parameters were evaluated on univariate analysis by the chi-square (chi^2^) statistic for their correlation to disease progression or death from any cause. N: total number of patients; PD: progressive disease; *p*-val: *p*-value; D: death of any cause; PS: performance status; ECOG: Eastern Cooperative Oncology Group; MSK: Memorial Sloan-Kettering Cancer Center; IESLG: international extranodal lymphoma study group; LDH: serum lactate dehydrogenase; GC: germinal center type histology; non GC: non germinal center type histology; PET2–: negative PET2 result; PET2+: positive PET2 result.

## DISCUSSION

In this current study, we assessed the prognostic value of PET2 in a series of 25 newly diagnosed immunocompetent PCNSL patients treated by the RMT regimen and various consolidative strategies. We observed that PET2 strongly correlated to end-of-treatment MRI-assessed response, and to PFS. The prognostic value of early response assessment in PCNSL has seldom been evaluated, and current available data are contradictory [14, 15]. A recent analysis of MRI-defined response after the first line of chemotherapy did not report differences in the outcome of patients achieving an early CR (assessed two month after treatment onset) compared to those experiencing a delayed CR [15]. While the authors considered that the response kinetic had no prognostic value in PCNSL, this result may also be interpreted as a limit of MRI-assessed evaluation to discriminate CR patients at an early stage after initiation of therapy.

In striking contrast with other lymphoma subtypes, the role of PET has been marginally studied in PCNSL [13]. As a diagnostic tool, PET may contribute to discriminating PCNSL from other malignant brain tumors, though an histological confirmation remains mandatory [16, 17]. Importantly, PET is widely used to distinguish systemic lymphoma with CNS involvement from PCNSL. Indeed, a study reported that as much as 7% of presumed PCNSL patients had extra-CNS involvement found by PET, which was unapparent after full-body CT/scan and bone marrow biopsy [18]. Moreover, two studies reported a potential prognostic application of PET initial maximum Standard Uptake Value (SUVmax) in PCNSL [19, 20]. Similar to our current analysis, a retrospective study found that end-of-treatment – but not interim – PET correlated to PFS [21]. While requiring extensive confirmative studies in homogeneously treated PCNSL patients, we believe that these reports, along with our current data, pave the way for integrating PET into therapeutic strategies in this disease.

We attempted to analyze initial metabolic tumor volume and SUVmax, but our interpretation was biased by the frequent administration of corticosteroids before initial PET evaluation [17]. Despite this limitation, initial PET displayed a pathological 18F-FDG uptake in all evaluable patients. Focusing on interim and post-treatment PET, we observed no discrepancies between PET and MRI evaluations in CR and SD/PD patients. Interestingly, 5 PET2- patients had a MRI PR after two RMT cycles, and all of them subsequently reached end-of-treatment CR. This suggested that PET may more accurately distinguish between responding and non-responding patients than the IPCG radiographic response criteria at early stages of therapy. The growing availability of new metabolic markers such as choline isotopes may further enhance the sensitivity of PET in PCNSL patients in the future.

Although PET2- seems to be predictive of a favorable outcome based on PFS analysis, this test had no impact on OS in our study. We hypothesized that this may result from our seemingly limited follow-up (29 months) that may not capture all disease-related events, but also to the efficient salvage strategies, including ASCT, used in progressive or relapsing patients. In our study, among 7 refractory/relapsing patients, 3 achieved long-term CR after salvage therapy and autologous stem cell transplantation. Moreover, age over 60 and PS were not correlated to response or survival in our analysis, in contrast to other studies. We hypothesized that a limited follow-up period and small sample size might account for this discrepancy as well.

Our results show, for the first time, that early PET evaluation may be useful in PCNSL intensively treated by Methotrexate-based combination protocols. More particularly, PET may better identify early responders than MRI, suggesting that future risk-stratified therapeutic strategies might take advantage of PET evaluation to identify patients requiring frontline intensification or de-escalation, aiming for optimal, personalized medicine strategies in this frequently fatal disease.

## MATERIALS AND METHODS

### Study design and patient selection

The study protocol employed a retrospective, consecutive entry design. We evaluated all the patients (*n* = 28) consecutively treated for newly-diagnosed PCNSL from November 2013 to December 2016 at a single tertiary-care university hospital. Three patients were excluded due to the absence of PET2 evaluation, including one technical failure (hyperglycemia), one early death after the first course of chemotherapy in the absence of PET or MRI evaluation and one early PD who did not underwent PET2 evaluation. We used the 2008 World Health Organization classification for PCNSL diagnosis. All cases were centrally reviewed for histology. All patients were enrolled into the French oculo-cerebral lymphoma network (LOC) register. Contrast-enhanced magnetic resonance imaging (MRI) was performed at diagnosis for all patients. Ophthalmological evaluation and cerebrospinal fluid (CSF) cytology were performed at the time of diagnosis or during therapy. Five patients underwent a complete surgical resection of their tumor before PCNSL diagnosis.

### Treatment

Induction treatment consisted of four cycles of 375 mg/m^2^ Rituximab (days 1 and 15), 8 g/m^2^ Methotrexate (days 1 and 15) and Temozolomide 150 mg/m^2^ (days 7 through 11), repeated every four weeks (the RMT regimen). Corticosteroids, which were used during the perioperative period in nearly all patients, were tapered within few days after RMT onset. A consolidation therapy was given at the discretion of physician, dependent on age and comorbidities. We used intravenous Etoposide 40 mg/kg on a continuous 96 h fusion days 1 through 4 and Aracytine 2 g/m^2^ twice a day days 1 through 4 (the EA regimen) in 13 patients; whole brain radiotherapy at 30.6 Gy in 1 patient and 23.4 Gy in 3 patients; and no consolidation in 2 patients. Patients with stable or progressive disease during induction were switched to various salvage therapies.

### Response assessment

Brain MRI and PET were performed at diagnosis, after two and four cycles of RMT, and after treatment completion (ie. after EA or radiotherapy consolidation, or after four cycles of RMT in the absence of consolidation). The MRI protocol included FLAIR, T1 and gadolinium enhanced T1 sequences. Response was evaluated according to IPCG criteria [12]. Notably, PET results neither had influence on response assessment nor resulted in treatment modification. Briefly, complete response (CR) was defined as complete disappearance of all enhancing abnormalities on gadolinium-enhanced MRI. CR/unconfirmed (CRu) was defined by CR with persistent enhancing abnormality on MRI related to biopsy or focal hemorrhage. Partial response (PR) was defined as a greater than 50% decrease in the MRI contrast-enhanced lesion. Progressive disease (PD) was defined as a more than 25% increase in the MRI contrast-enhancing lesion, or/and the appearance of new lesions. Any other situation was considered as stable disease (SD). If once found positive, CSF and/or slit lamp evaluation were repeated at the end of treatment. MRI evaluations were performed on routine basis at a single tertiary-care neurological center, and were subsequently reviewed by a single reader; leading to the reclassification of two PD patients to SD. MRIe (MRI end of treatment) was performed at end of the first line therapy. In patients with PD or SD, MRIe referred to the first evaluation showing PD or SD (see for example Table 3: MRI2 for patients 22-23-24 or MRI4 for patient 21). In responders, MRIe could be MRI4 (if no consolidation, eg. patient 15), or later MRI evaluations (eg. patient 14).

### PET protocol (Figure 3)

Whole-body PET images were obtained using a PET system (Discovery 690; GE Healthcare) and were acquired at a single institution. The protocol involved the combination of a full-ring PET scanner with Bismuth Germanium Oxide (BGO) crystals and a 4-row helical CT scanner. Patients fasted for at least 4 h prior to administration of 3 MBq/kg of 18F-FDG. Following body scan at 60 min from the skull base to the upper thigh, brain acquisitions were done 80 min after injection using the following parameters for CT (120 kV, 200 mAs, field of view 70 mm and slice thickness of 2,5 mm) without concomitant administration of an intravenous contrast agent. PET response was binary assessed based on a visual analysis. Positive PET was defined by a new brain lesion, or the persistence of 18F-FDG uptake compared to the contralateral parenchyma, while negative PET corresponded the strict absence of tumor metabolism. PET evaluations were performed in clinical routine, and all cases were then submitted to a blind review by a single reader. No discrepancies were observed between these evaluations.

**Figure 3:**
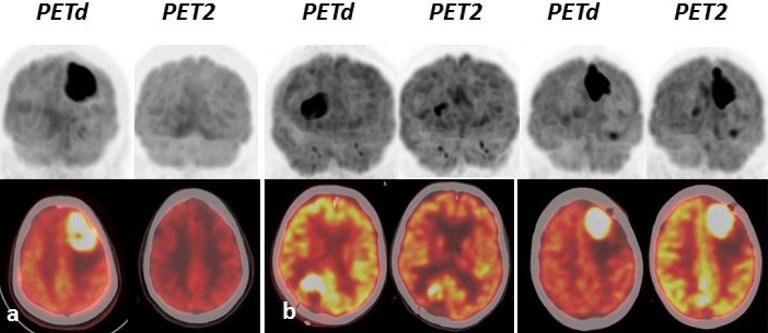
Three illustrative cases of PET images showing negative (**A**) and positive (**B**) PET2. PETd: PET at diagnosis; PET2: PET after 2 RMT cycles.

### Statistical analysis

Progression-free survival (PFS) was defined as the time from diagnosis until progression, relapse from CR, or death. Overall survival (OS) was defined as the time from diagnosis until death from any cause. Survival was assessed by the Kaplan and Meier method and the prognostic factors were compared using the log-rank test. Considering the limited numbers of patients, multivariate analysis was not performed. Association between PET2 and end of treatment response was evaluated using Chi-square (Chi^2^) test. Statistical analysis was carried out with XLSTAT.

## Abbreviations

ASCT: Autologous stem cell transplantation
CR: Complete response
CRu: Complete Response unconfirmed
ETR: End of treatment response
IESLG: International Extranodal Lymphoma Study Group experience
MRI: Magnetic resonance imagery
MSK: Memorial Sloan-Kettering
NE: Non evaluated
OS: Overall survival
PCNSL: Primary central nervous system lymphoma
PD: Progressive Disease
PFS: progression free survival
PR: Partial Response
RMT: Rituximab Methotrexate Temozolomide
SD: Stable disease
WBRT: Whole brain radiotherapy
